# Alternative Mechanism for PFOA?: Trout Studies Shed Light on Liver Effects

**Published:** 2008-08

**Authors:** Ernie Hood

Perfluorooctanoic acid (PFOA), used to make a class of industrial chemicals that are widely used in products such as textile coatings and flame retardants, is known to be a potent hepatocarcinogen in rodents. Until now the only mechanism of action for PFOA identified in rodent studies has been peroxisome proliferation, a well-characterized form of oxidative stress. Humans are reportedly insensitive to peroxisome proliferation; however, concerns remain that PFOA may cause adverse effects in people as well as in laboratory animals. Using rainbow trout as a model for chemically induced liver cancer in humans, a team of researchers suggest a new mechanism for the carcinogenicity of PFOA that does not involve peroxisome proliferation **[*EHP* 116:1047–1055; Tilton et al.]**.

The investigators compared the hepatocarcinogenic potential of PFOA against the structurally diverse peroxisome proliferators cloribrate (CLOF) and dehydroepiandrosterone (DHEA), identifying mechanisms of carcinogenesis from hepatic gene expression profiles phenotypically anchored to tumor outcome. Trout were fed PFOA in the diet for 30 weeks for tumor analysis. The investigators subsequently examined gene expression by cDNA array in animals fed PFOA, DHEA, CHOF, or 17β-estradiol (E_2_; a known tumor promoter) in the diet for 14 days.

The study showed that PFOA and DHEA treatments significantly increased liver tumor incidence and multiplicity; CLOF showed no effect. However, carcinogenesis was independent of peroxisome proliferation as measured by the lack of peroxisomal β-oxidation and catalase activity. On the contrary, both PFOA and DHEA resulted in estrogenic gene signatures closely resembling that of E_2_. CLOF, however, regulated no genes in common with E_2_.

Although the current study did not identify a threshold for the estrogenic effect of PFOA, the results indicate that PFOA is weakly estrogenic in trout and that its association with trout liver cancer may be related to disruptions in estrogenic signaling. More studies are needed to assess the potential for PFOA-mediated carcinogenesis in other species that are insensitive to peroxisome proliferation. Considering the mechanism identified in this study, the consequences of hormone-related effects by PFOA should be evaluated in other tissues, models, and sensitive life stages.

## Figures and Tables

**Figure:**
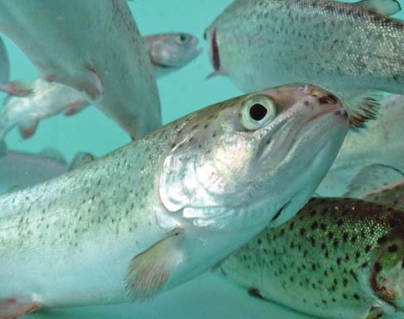
Mt. Shasta strain rainbow trout (*Oncorhynchus mykiss*)

